# A Decentralized Approach to the Formulation of Hypotheses: A Hierarchical Structural Model for a Prion Self-Assembled System

**DOI:** 10.1038/srep30633

**Published:** 2016-07-28

**Authors:** Mingyang Wang, Feifei Zhang, Chao Song, Pengfei Shi, Jin Zhu

**Affiliations:** 1 Department of Polymer Science and Engineering, School of Chemistry and Chemical Engineering, State Key Laboratory of Coordination Chemistry, Nanjing National Laboratory of Microstructures, Collaborative Innovation Center of Chemistry for Life Sciences, Nanjing University, Nanjing 210093, China

## Abstract

Innovation in hypotheses is a key transformative driver for scientific development. The conventional centralized hypothesis formulation approach, where a dominant hypothesis is typically derived from a primary phenomenon, can, inevitably, impose restriction on the range of conceivable experiments and legitimate hypotheses, and ultimately impede understanding of the system of interest. We report herein the proposal of a decentralized approach for the formulation of hypotheses, through initial preconception-free phenomenon accumulation and subsequent reticular logical reasoning processes. The two-step approach can provide an unbiased, panoramic view of the system and as such should enable the generation of a set of more coherent and therefore plausible hypotheses. As a proof-of-concept demonstration of the utility of this open-ended approach, a hierarchical model has been developed for a prion self-assembled system, allowing insight into hitherto elusive static and dynamic features associated with this intriguing structure.

The ultimate goal of any scientific endeavor is to elucidate the working principle of a system and use that understanding to benefit mankind. To this end, a centralized method (Fig. 1)^1^, which utilizes a primary phenomenon to derive a dominant hypothesis for a local aspect of the system, prevails in the scientific community. The major drawback of this close-ended, essentially *ad hoc* approach is that it can impose restriction on the range of conceivable experiments, thwart effort to seek alternative legitimate hypotheses, and ultimately impede understanding of the system. A global strategy (such as “omics”^2^,^3^,^4^) aims to tackle the whole puzzle through systemic scrutiny and integrated analysis of the entire network. This inspiring conceptual framework, albeit fascinating, can be technically extremely challenging given the inundating, vast complexity of the totality of patterns to be entertained. We believe that a more constructive hypothesis formulation method should be locally focused yet sufficiently decentralized (accumulation of a broad suite of primary phenomena without preconception of any kind, compatible with or in contradiction to the currently accepted scientific principles) (Fig. 1). This open-ended, decentralized approach can provide a more panoramic view of the target aspect of the system, and the reticular network of phenomena should enable the formulation of a set of more coherent and therefore plausible hypotheses. Indeed, in contrast to the centralized approach, where many-to-one correspondence (many phenomena, primary and secondary/in series, to one hypothesis) is the key characteristic, the decentralized approach allows the simultaneous derivation of many hypotheses based on comprehensive many-to-many logical reasoning from phenomena to hypotheses (in parallel). To turn decentralized approach into an effective research tool, one needs to meticulously dissect experimental results, through cross-correlation when necessary, into phenomena, and use those fundamental rendition units for inductive/deductive reasoning analysis and generation of hypotheses. The system can then be rationalized, if desired, by seamless overlapping of consistent hypotheses for different regional aspects of the system, which can be likened to the description of a mathematical manifold using the chart-atlas structure^5^.

## Results and Discussion

### General

**Figure 2:**
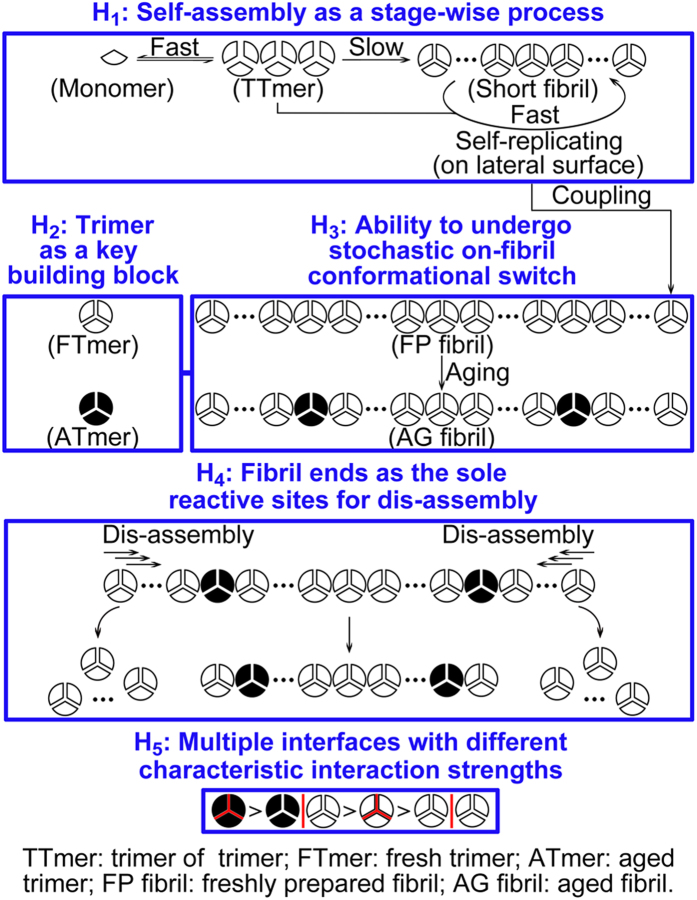
Schematic diagram of five hypotheses derived, through the decentralized approach, for a prion self-assembled system.

Herein, through this decentralized approach, we wish to report the proposal of a hierarchical structural model for a prion self-assembled system. In particular, we seek insight into the self-assembly aspect of prion by perturbation and observation of the system without *a priori* target hypothesis in mind. The suite of phenomena compiled from static and dynamic analysis of prion fibril allows the revelation of hitherto elusive structural features for this self-assembled system (Fig. 2): 1) self-assembly as a stage-wise process, 2) prion trimer as a key building block, 3) ability to undergo stochastic on-fibril conformational switch, 4) fibril ends as the sole reactive sites for dis-assembly, and 5) existence of multiple interfaces with different characteristic interaction strengths.

Prion is a class of intriguing protein species that has significant implications in chemistry^6^,^7^,^8^, biology^9^,^10^, and medicine^11^,^12^,^13^,^14^. Extensive efforts worldwide have been devoted to the study of its self-assembly mechanism^15^,^16^,^17^ and architecture^18^,^19^,^20^,^21^. In spite of the progress, no detailed structural model has been proposed based on the centralized approach. We have recently launched a research program on this type of self-propagating entity using Sup35-NM, a protein segment comprising N-terminal and middle domains of the yeast *S. cerevisiae* prion Sup35, as the model prion system^22^,^23^,^24^. Self-assembly (fibril growth) and dis-assembly (perturbation of fibrils with disruption forces) processes have been closely monitored for the development of a detailed structural model. Fibril growth is initiated by removal of urea, a denaturing agent, in the Sup35-NM stock solution through a desalting column. The self-assembly then proceeds at 4 °C and at the end point, three types of fibrils have been generated in this investigation: freshly prepared (FP) fibrils, aged (AG) fibrils, and stirring-yielded (ST) fibrils. FP fibrils refer to fibrils self-assembled for 1 week; AG fibrils refer to fibrils produced through self-assembly for over 1 month; ST fibrils refer to fibrils created via the additional participation of stirring force. The disruption forces for dis-assembly include sodium dodecylsulfate (SDS), boiling, and electric field. Size exclusion chromatography (SEC)^25^, blue native polyacrylamide gel electrophoresis (BN-PAGE)^26^, Congo red binding experiment^27^, transmission electron microscopy (TEM)^28^, and western blot assay^29^ are used as experimental tools for analysis.

### Hypothesis 1

**Figure 3:**
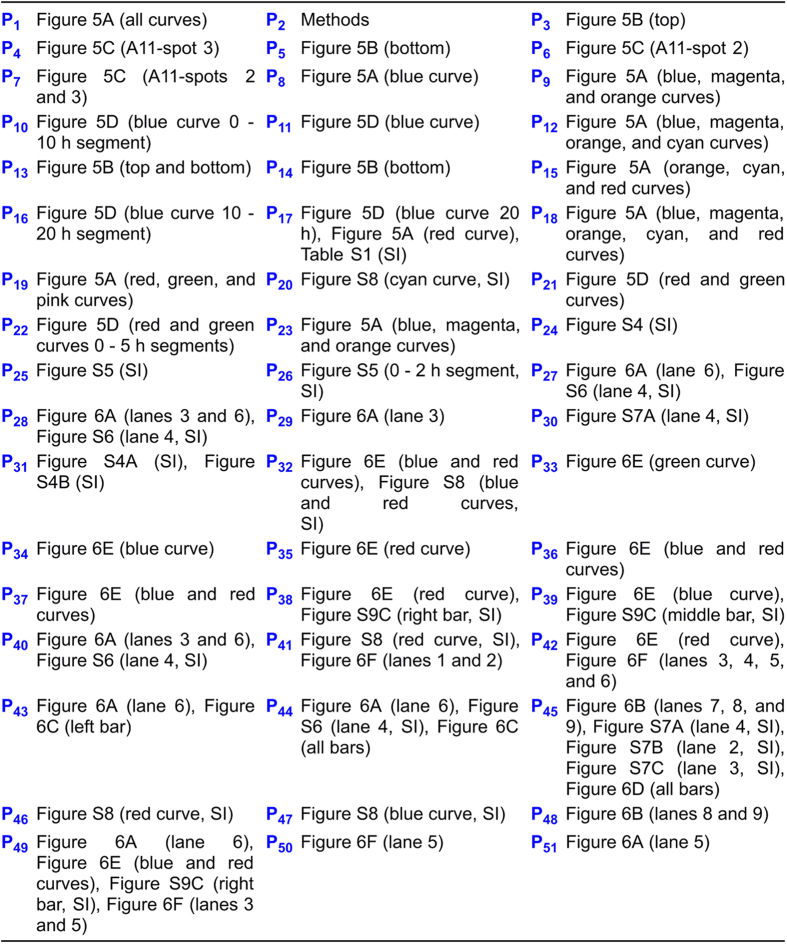
Dissection of experimental results into phenomena.

**Figure 4:**
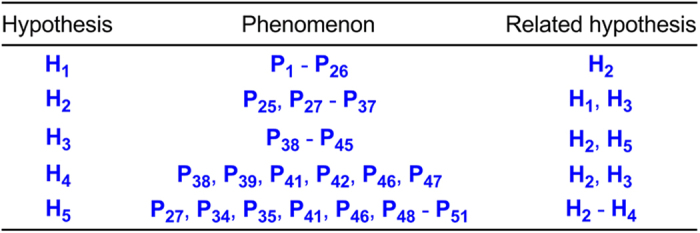
Relationship between phenomena and hypotheses.

**Figure 5:**
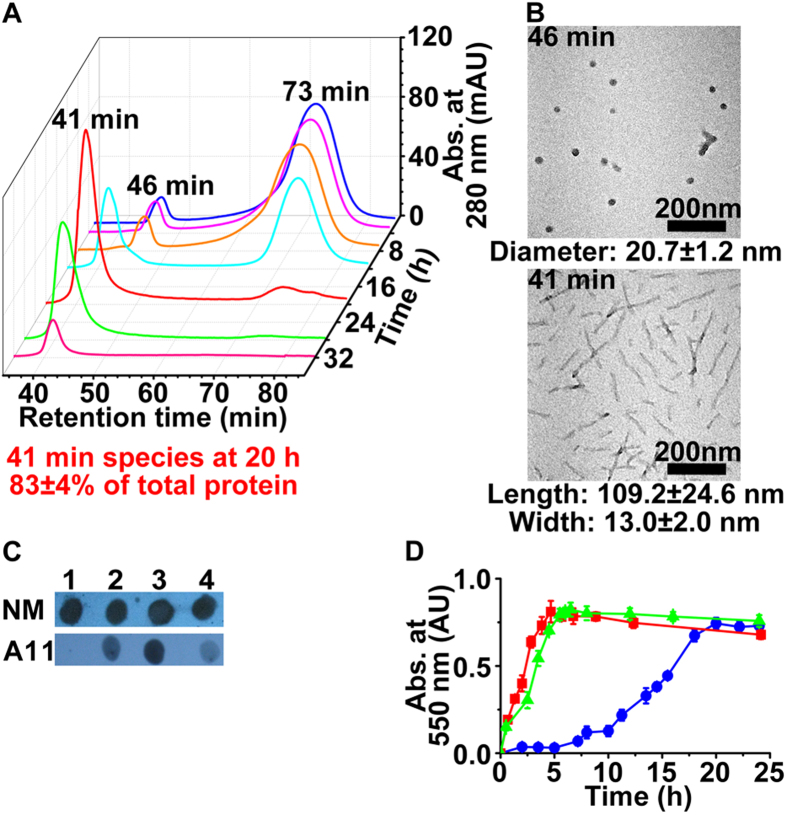
Self-assembly process for Sup35-NM. (**A**) Time-course tracking of Sup35-NM self-assembly process as monitored by SEC. The Sup35-NM solution was analyzed at 2 h (blue), 4 h (magenta), 8 h (orange), 12 h (cyan), 20 h (red), 28 h (green), and 32 h (pink). (**B**) TEM images of 46 min (size determined from 50 spherical-shaped structures) and 41 min (size determined from 100 fibrils) species from SEC. (**C**) Western blots for Sup35-NM monomer (in 8 M urea, spot 1) and 41 min (spot 2), 46 min (spot 3), 73 min (spot 4) species from SEC. An anti-Sup35-NM, sequence-specific antibody was used for the NM lane. An oligomer-specific antibody was used for the A11 lane^21^,^25^,^29^. (**D**) Sup35-NM self-assembly process, as monitored by Congo red binding experiment, in the absence (blue) and presence of short fibril (red) or FP fibril (green).

**Figure 6:**
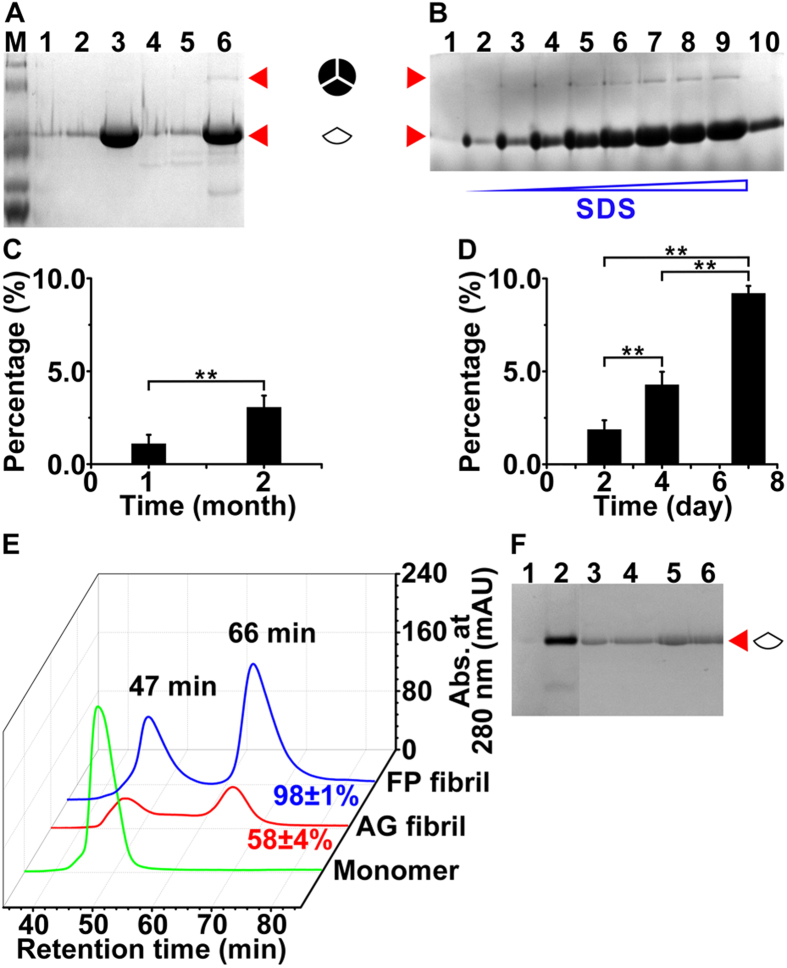
Dis-assembly studies of Sup35-NM fibrils. (**A**) Electrophoregram for FP and AG fibrils under SDS treatment. Lane 1: directly loaded FP fibril; lane 2: FP fibril under SDS; lane 3: FP fibril under SDS-boiling; lane 4: directly loaded AG fibril; lane 5: AG fibril under SDS; lane 6: AG fibril under SDS-boiling; lane M: molecular weight protein marker comprising 116 kDa, 66.2 kDa, 45 kDa, 35 kDa, 25 kDa, and 18.4 kDa. (**B**) Electrophoregram for ST fibril (2-day) under SDS treatment. Lane 1: directly loaded ST fibril; lanes 2–9: ST-fibril under SDS-boiling (0.55%, 1.1%, 1.65%, 2.2%, 3.3%, 4.4%, 5.5%, 6.6%); lane 10: ST-fibril under SDS (6.6%). (**C**) Percentage of ATmer in 1-month (Fig. 6A, lane 6) and 2-month (Figure S6, lane 4) AG fibrils. Student’s t-test: **P < 0.01 (mean ± s.d.). (**D**) Percentage of ATmer in 2-day ST fibril after being stored at 4 °C for different number of days (0 day: Fig. 6B, lanes 7, 8, 9 or Figure S7A, lane 4; 2 days: Figure S7B, lane 2; 5 days: Figure S7C, lane 3). Student’s t-test: **P < 0.01 (mean ± s.d.). (**E**) SEC curves for FP fibril (blue), AG fibril (red), and Sup35-NM monomer (green) under SDS-boiling treatment. (**F**) Electrophoregram for AG fibril under SDS treatment. 41 min species (Figure S8, red): directly loaded (lane 1) and under SDS-boiling (lane 2); 47 min species (Fig. 6E, red): directly loaded (lane 3) and under SDS-boiling (lane 4); 66 min species (Fig. 6E, red): directly loaded (lane 5) and under SDS-boiling (lane 6).

The first hypothesis, self-assembly as a stage-wise process (Figs 2, 3 and 4), is formulated through the identification of intermediate species and observation of fibril growth dynamics: Time-course tracking of fibril generation process by SEC reveals a fast equilibrium between monomer and trimer of trimer (TTmer, an intermediate species), a slow conversion of TTmer to short fibril, and a fast self-replicating of short fibril through the consumption of TTmer. With the removal of urea from the Sup35-NM stock solution, one can identify several distinct peaks on SEC with the evolution of time: 73 min, 46 min, and 41 min (**P**_**1**_; for the dissection of experimental results into phenomena, see Fig. 3; for the relationship between phenomena and hypotheses, see Fig. 4; for the main experimental results, see Figs 5 and 6). The 73 min peak can be obviously assigned as renatured monomer. The 46 min peak is assigned as TTmer because of the following lines of evidence: i) the identification of trimer band on BN-PAGE (see second hypothesis, *vide infra*); ii) a similar retention time (45 min) for a standard globular protein, ferritin (12 nm, 440 kDa) (**P**_**2**_)^30^; iii) the observation of spherical-shaped structure under TEM for a 46 min sample (**P**_**3**_); iv) confirmation of the presence of oligomeric motif in the 46 min solution via western blot analysis by a prion oligomer-specific antibody (**P**_**4**_). The 41 min peak is assigned as short fibril based on TEM identification of such a structure (**P**_**5**_). Western blot assay again confirms the presence of oligomeric structure in the short fibril (**P**_**6**_), and its lower intensity compared with TTmer (**P**_**7**_) signifies further (partial) burial of oligomeric epitope determinant, most likely inside the fibril instead of at the fibril ends. The existence of a fast equilibrium between monomer and TTmer is inferred from: i) a fast emergence of TTmer on SEC (**P**_**8**_); ii) co-existence of monomer and TTmer with virtually identical composition (SEC) (**P**_**9**_) throughout the incubation period (0 to 10 h) (**P**_**10**_) in the S-shaped self-assembly dynamics curve (Congo red binding experiment) (**P**_**11**_)^15^,^31^. The emergence of short fibril from TTmer is a slow process (**P**_**12**_), corresponding to the incubation period (**P**_**10**_). The diameter of TTmer (21 nm) is larger than the width of short fibril (109 nm in length, 13 nm in width) (**P**_**13**_). The lack of individually identifiable spherical-shaped structure in the short fibril (**P**_**14**_) suggests its role as the source of building block instead of as a nucleation/catalysis seeding site. Further, dimensional shrinkage of TTmer upon self-assembly into short fibril (**P**_**13**_) suggests the tightening of intra-trimer interface during this process. The fast short-fibril self-replicating process (**P**_**15**_) corresponds to the accelerated growth stage (10 to 20 h) (**P**_**16**_) in S-shaped curve and at the end of this stage, 41 min species accounts for the majority of total protein (83%) (**P**_**17**_). Although TTmer cannot be observed on SEC during this process, it is the authentic structural motif that self-assembles into the short fibril. Indeed, if monomer is directly converted into short fibril, the 46 min TTmer peak should not disappear right after the start of accelerated fibril growth (**P**_**15**_) since it will remain in equilibrium with monomer (**P**_**9**_). Further, templated conversion from TTmer to short fibril occurs at a rate faster than the conversion of monomer to TTmer (**P**_**18**_). Most likely, the lateral surface of fibril is the catalytic site for the occurrence of self-replicating process: 1) The S-shaped exponential growth (**P**_**11**_) requires a self-replicating mechanism; 2) Fibril breakage is not observed in our system (**P**_**19**_, **P**_**20**_), in contradiction to the end growth proposition; 3) Fibril growth can be accelerated by both short and long (FP) fibrils to a comparable extent (**P**_**21**_), and no incubation period is observed (**P**_**22**_); 4) Preliminary experiment indicates the absence of acceleration effect from TTmer (**P**_**23**_), and the major structural difference between fibril and TTmer is the apparently expanded lateral surface area in fibril (**P**_**3**_–**P**_**7**_, **P**_**13**_, **P**_**14**_). Eventually, the SEC peak at 41 min is decreased (**P**_**19**_) as the short fibrils react with one another (via coupling at fibril ends) to form longer fibrils (**P**_**24**_), the size of which prevents identification on the SEC facility employed herein. The stirring action can substantially accelerate the fibril growth from monomer, allowing the finishing of growing process within 2 h (**P**_**25**_), again without any incubation period (**P**_**26**_). Apparently, via increase of the collision probability, stirring promotes the initial conversion of TTmer into fibril and subsequent fibril self-replicating process.

### Hypothesis 2

The second hypothesis, prion trimer as a key building block (Figs 2, 3 and 4), is derived from several observations: 1) Under SDS-boiling condition, AG fibrils exhibit a unique BN-PAGE band at 100 kDa (**P**_**27**_) besides the monomer band (calculated as 37 kDa from BN-PAGE, as 31 kDa from amino acid sequence) (**P**_**28**_). The molecular weight is approximately three times that of the monomer and as such the band is assigned as aged trimer (ATmer). This aging-derived higher molecular weight band is not an artifact since FP fibrils exhibit, exclusively, monomer band (on BN-PAGE) (**P**_**29**_) under otherwise identical conditions. In fact, stirring can substantially accelerate the emergence of ATmer (**P**_**30**_) in ST fibrils (**P**_**25**_); 2) The ATmer most likely originates from a trimer (fresh trimer, or FTmer) unit in the FP fibrils, through an on-fibril conformational switch (see third hypothesis, *vide infra*) and therefore tightening of the intra-trimer interface. Indeed, the structural reorganization for an on-fibril monomer-to-trimer conformational switch should cause dimensional change in the fibril radial direction, which is not observed under TEM (**P**_**31**_), and is believed to be thermodynamically too costly; 3) SDS can contribute to both of the prion dis-assembly and self-assembly processes (**P**_**32**_–**P**_**37**_). SDS can cause the dissociation of trimer (FTmer) units from longer (AG and FP) fibrils and simultaneously accelerate the arrival of equilibrium among different-sized prion species (**P**_**32**_). The SEC elution patterns for monomer forward self-assembly (**P**_**33**_) and fibril (FP and 1-month AG) backward dis-assembly (**P**_**34**_, **P**_**35**_) under SDS-boiling condition are different, suggesting that there exists, in the fibril, a robust building block larger than the monomer unit. Considering the absence of peak between 66 min and 73 min (**P**_**36**_), the 66 min species is assigned as the trimer unit, the dissociated robust building block that survives the SDS-boiling condition. Obviously, SDS can promote further self-assembly of this trimer unit into TTmer (47 min peak) (**P**_**37**_).

### Hypothesis 3

The third hypothesis, ability to undergo stochastic on-fibril conformational switch (Figs 2, 3 and 4), is closely related to the second hypothesis. The stochastic nature of the conformational switch process is supported by the following observations: 1) ATmer is distributed in a random instead of clustered manner. The dissociated portion of 1-month AG fibrils (58% by SEC) (**P**_**38**_) is substantially smaller than that of FP fibrils (98%) (**P**_**39**_) under SDS-boiling condition, suggesting the existence of a stop point for the dissociation process on AG fibrils. The major difference between AG fibrils and FP fibrils is the existence of ATmer (**P**_**40**_) and as such ATmer/FTmer interface is the only candidate for stop point. In support of this, no ATmer band has been identified on BN-PAGE for a dissociated portion of AG fibrils (under SDS or SDS-boiling condition) collected from SEC eluted solution (**P**_**41**_, **P**_**42**_). The percentage of ATmer in the whole AG fibril is only 1.1% according to BN-PAGE (**P**_**43**_) and therefore only random distribution of ATmer allows the generation of required number of ATmer/FTmer interfaces; 2) No apparent acceleration in the increase of ATmer percentage occurs over time, under both static (**P**_**44**_) and stirring (**P**_**45**_) conditions, suggesting that the FTmer-to-ATmer conformational switch process is spontaneous and not induced by neighboring ATmer.

### Hypothesis 4

The fourth hypothesis, fibril ends as the sole reactive sites for dis-assembly (Figs 2, 3 and 4), is proposed based on the following phenomena: 1) The absence of ATmer band on BN-PAGE for SEC eluted, dissociated AG fibril sample (**P**_**41**_, **P**_**42**_) excludes the possibility of fibril scission at random sites; 2) The random scission should lead to virtually identical dissociated portion of AG and FP fibrils on SEC, which is not observed (**P**_**38**_, **P**_**39**_, **P**_**46**_, **P**_**47**_). The exact dis-assembly stop point should be, starting from the fibril ends, the first FTmer encountered neighboring to an ATmer.

### Hypothesis 5

The fifth hypothesis, existence of multiple interfaces with different characteristic interaction strengths (Figs 2, 3 and 4), is derived from a diverse set of phenomena: 1) The intra-trimer interface of ATmer is the strongest since it is the only one that survives both SDS-boiling condition and electric field (BN-PAGE) (**P**_**27**_). Such an interface is stable even under an elevated concentration of SDS (**P**_**48**_); 2) The AG fibril dissociation stop point under SDS-boiling condition is ATmer/FTmer interface (see third hypothesis, *vide supra*), whereas all the FTmer/FTmer interfaces located between the fibril ends and ATmer/FTmer interface are disrupted (**P**_**49**_), suggesting that ATmer/FTmer interface is stronger than FTmer/FTmer interface; 3) FTmer is not dissociated into monomer units under SDS-boiling condition (**P**_**34**_, **P**_**35**_) but dissociated under electric field (**P**_**50**_), providing evidence that intra-trimer interface of FTmer is stronger than FTmer/FTmer interface; 4) The percentage of dissociated AG fibrils under SDS condition is 46% (**P**_**46**_) and no ATmer band is observed on BN-PAGE (**P**_**41**_), indicating that ATmer/FTmer interface remains intact. On the other hand, only 13% of monomer band appears on BN-PAGE (**P**_**51**_) and therefore we can infer that ATmer/FTmer interface is intact whereas intra-trimer interface of FTmer is disrupted. Taken together, ATmer/FTmer interface is stronger than the intra-trimer interface of FTmer.

## Conclusion

In summary, we have proposed a decentralized hypothesis formulation approach and use that approach to develop a hierarchical model for a prion self-assembled system. Unprecedented insight has been gained into both static and dynamic aspects of this intriguing structure through the combination of bottom-up (self-assembly) and top-down (dis-assembly) analysis strategies. The centralized hypothesis formulation approach has dominated scientific thinking and practice for many centuries. The dominant hypothesis expeditiously generated from a primary phenomenon can sometimes be misleading as a result of limited information. Indeed, with the adoption of decentralized approach, the expansion of experimental scope can in certain cases allow the exposure of otherwise hidden conflicting phenomena and therefore fixing of previously erroneous hypothesis. More importantly, not all scientific hypotheses can be formulated through the centralized approach. The primary phenomenon can be elusive for observation, due to, for example, its transient nature. Under this circumstance, logically equivalent suite of phenomena can sometimes be manifested in the decentralized context for the establishment of identical hypothesis. Further, the decentralized approach can contribute to the derivation of more highly sought after “central dogmas” through creative, intertwined imagination of both global rules and local postulates (restrictions/categorizations) under the framework of logical compatibility. Taken together, it is expected that, with an ever increased demand for comprehensive understanding of Nature’s complex working principles, the decentralized approach proposed herein should become the method of choice for future scientific investigations.

## Methods

### Strains and culture

*Sup35-NM* was cloned from 74-D694 [*psi*^−^] genomic DNA. Restriction endonuclease digestion sites, Not I and Nde I, were chosen as *Sup35-NM* insert sites for pET28a expression vector. The primers’ sequences were: Sup35-NM-Forward, 5′-ATGGGTCGCGGATCCGAATTCATGTCGGATTCAAAC CAAGGC-3′; Sup35-NM-Reverse, 5′-TGGTGGTGCTCGAGTGCGGCCGCATCGTTAACAACTTCGTCATCC-3′. The target pET28a-Sup35-NM vector was amplified in DH5*α* (transformed by traditional heat-shock transfer method) and sequenced for the verification of as-designed sequence. BL21 Rosseta (DE3) cells harboring the pET28a-Sup35-NM vector were used for the expression and purification of Sup35-NM. The transformants were cultured in LB medium with 50 mg/L kanamycin at 37 °C and 220 rpm. When these cells’ OD_600_ reached 1.0, IPTG was added to the medium with a final concentration of 0.5 mM. The growing condition for these transformants was then switched to 30 °C and 160 rpm. After 4 h, the cells were harvested at 4 °C and 5000 g for 10 min.

### Purification of denatured Sup35-NM protein

The induced cells for Sup35-NM were resuspended in lysis buffer (20 mM Tris, pH 8.0, 8 M urea, 100 mM NaCl). Bacterial suspension was then sonicated for cell lysis. The 50 mL lysis mixture from 1.5 L total culture was centrifuged at 8000 g for 10 min and the supernatant was filtered by 0.22 μm membranes. Ni^2+^-NTA agarose column (3 cm × 8 cm, 10 mL packing) was used for the first-stage purification. The column was washed with wash buffer (20 mM Tris, pH 8.0, 8 M urea, 20 mM imidazole, 100 mM NaCl) for 20 column volumes (flow rate 0.5 mL/min). The target protein was eluted by 250 mM imidazole (20 mM Tris, pH 8.0, 8 M urea, 250 mM imidazole, 100 mM NaCl).

The following purification was conducted with a GE AKTA purifier (pH/c-900, UV-900, P-900, frac-900) at 4 °C. Anion-exchange chromatography was used for the second-stage protein purification. The purification was conducted at 4 °C and 4 M urea, instead of 8 M, was used because 8 M urea was unstable and could form precipitates at a low temperature. The target protein eluted from the Ni^2+^-NTA agarose column was pumped into a Q HP prepacking column (3 mL/min, 17-1154-01). The column was washed with a stepped gradient of two buffers (buffer A: 20 mM Tris, pH 8.0, 4 M urea; buffer B: 20 mM Tris, pH 8.0, 4 M urea, 300 mM NaCl) for 20 column volumes (flow rate 3 mL/min). The 50% gradient target fraction was concentrated by ultrafiltration (Millipore, Amicon ultra centrifugal filters) and then injected into a HiLoad Superdex 200 pg 16/60 size exclusion chromatographic column (1 mL/min, 17-1069-01) for the last-stage purification (flowing phase: buffer A). An important note is that the size exclusion chromatographic column must be washed with a cleaning-in-place (CIP, regular cleaning) protocol according to the instruction before use. The purified protein must be used immediately for preparing prion fibrils. Protein concentration was measured by BCA protein assay kit (Pierce, product #23227).

### Preparation and SEC analysis of Sup35-NM fibrils

Freshly prepared Sup35-NM denatured monomer (in 4 M urea) was desalted with buffer C (20 mM Tris, pH 8.0, 100 mM NaCl) by a HiTrap desalting column (5 mL, 17-1408-01) to remove urea and this was counted as the starting point for the growth of Sup35-NM fibril. The total sample was stored at 4 °C and the concentration was calculated by BCA method. Then, the same amount of sample was taken at different time intervals and injected into the size exclusion chromatographic column (HiLoad Superdex 200 pg 16/60) for the acquirement of fibril growth kinetics information and at the same time, used for Congo red binding assay. In order to get authentic Sup35-NM fibril growth kinetics information, it is extremely important that no fibrils or intermediates contaminate the desalting-prepared monomer. For this purpose, the desalting column must be used in a disposable format and the size exclusion chromatographic column must be washed with a flow rate of 25 cm/h (0.8 mL/min) at room temperature with the following steps (CIP, rigorous cleaning): first, 2 column volumes of 1 M NaOH followed by 4 column volumes of distilled water; second, 1.5 column volumes of 30% isopropanol followed by 2 column volumes of distilled water. All buffers in this part should be freshly prepared and all the processes were conducted at 4 °C except for CIP.

The gel phase distribution coefficient (K_av_) of Ferritin (440 kDa, Horse Spleen) in Gel Filtration Calibration kit LMW (28-4038-42) was close to 0.1, corresponding to a retention time of approximately 45 min, in HiLoad Superdex 200 pg 16/60 size exclusion chromatographic column (1 mL/min).

FP fibrils were obtained by standing at 4 °C for 1 week after the removal of 4 M urea by desalting. For the stirring protocol, desalted sample was divided into two parts: one was stored at 4 °C for the formation of FP fibrils (as a control), the other was stirred for 2 days with a 2 cm stir bar in 15 mL beaker at 4 °C and 800 rpm (2-day ST fibrils). The 2-day ST fibrils were then stored at 4 °C without stirring for the formation of 4-day (2 additional days) and 7-day (5 additional days) ST fibrils.

In the dis-assembly process, FP and AG fibrils (obtained after over 1 month) (20 μM) were boiled with 2% SDS for 5 min and then cooled down to room temperature. After 5 min, the samples were injected into the size exclusion chromatographic column for monitoring the dis-assembly process and all the SEC experiments were conducted at room temperature. When the BN-PAGE experiment was desired, the SEC-eluted SDS-treated fibrils were immediately loaded onto the BN-PAGE gel.

### TEM

The imaging-ready carbon-coated copper grid was placed on the sample and allowed to stay there for 3 min. The grid was then stained with a 2% uranyl formate aqueous solution (containing 25 mM NaOH) for 3 min, followed by twice washing with distilled water. TEM imaging was performed at 100 kV.

### Western blot assay

TBS-T buffer system (0.05% Tween-20) was used for all the western blot assay samples. The sample was applied onto a nitrocellulose membrane (PALL). After the membrane was dried, it was probed with anti-Sup35-NM sequence-specific antibody (Santa Cruz, sc25915) and amyloid oligomer-specific antibody (Millipore, AB9234)^21^. First, the membrane was blocked by 5% non-fat powered milk in TBS-T (5% non-fat powered milk/TBS-T, 1 h at room temperature). The membrane was then incubated with primary antibody (1:5000 dissolved in 5% non-fat powered milk/TBS-T) for 30 min at room temperature and was washed three times with TBS-T, each for 5 min. Then the secondary antibody (1:10000) conjugated with HRP was incubated on the membrane for 30 min at room temperature. Onto the membrane was loaded with an ECL solution (Pierce, product #34095) and after 1 min, X-ray film imaging was carried out in the dark room.

### Congo red binding experiment

The 10 mM Congo red stock buffer was filtered once by 0.45 μm membrane and twice by 0.22 μm membranes before use^32^. The final concentration used for all the binding experiments (Congo red as well as total protein of Sup35-NM) was 10 μM except that in Figure S3. The mixture was incubated for 30 min at 30 °C. Then, the 550 nm absorption was measured with a Bio-photometer plus facility (Eppendorf).

### BN-PAGE

The ingredients for the preparation of BN-PAGE gel were the same as those used for SDS-PAGE except in the absence of SDS. The basic (anode) buffer contained 20 mM Tris, 250 mM glycine. The cathode buffer contained an additional ingredient, 0.1% coomassie brilliant blue G250, besides those in the basic buffer. The sample eluted from the size exclusion chromatographic column did not need the addition of glycerol before being loaded onto the upper slots in the gel. The ATmer band could appear after the treatment of AG fibrils (20 μM) with 2% SDS under boiling condition for 5 min. At the beginning of the electrophoresis, an 80 V voltage was used for 20 min. Then 20 mA constant current was used throughout the rest of the process (for approximately 1.5 h). The gel could then be directly immersed in the destaining solution (distilled water:ethanol:acetic acid = 5:4:1) for 30 min. Then, the gel was further cleaned in water for almost 12 h to gain a clear view of the ATmer band.

### Calculation method for protein quantity and percentage associated with an elution peak from SEC

The areas of UV 280 nm absorption curves (fractional columnar area and the whole peak area) were integrated by Gel-Pro analyzer software. The protein quantity corresponding to the fractional columnar area was measured by BCA method. The protein quantity for the whole peak could then be calculated based on the method provided in Figure S9. The total protein before injection into the size exclusion chromatographic column was known through BCA method. Therefore, the percentage of protein associated with an elution peak over the total protein could be calculated.

### Statistical analysis

Data were presented as mean ± s.d. Statistical analyses were performed with SPSS 18.0 (SPSS Inc., USA). Paired student’s t-test was used and two-sided p-values less than 0.01 were considered statistically significant.

## Additional Information

**How to cite this article**: Wang, M. *et al*. A Decentralized Approach to the Formulation of Hypotheses: A Hierarchical Structural Model for a Prion Self-Assembled System. *Sci. Rep.* **6**, 30633; doi: 10.1038/srep30633 (2016).

## Supplementary Material

Supplementary Information

## Figures and Tables

**Figure 1:**
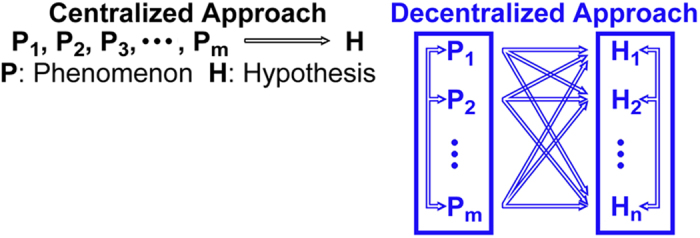
Schematic diagram of hypothesis formulation approaches: centralized (conventionally used) and decentralized (proposed herein). The centralized approach uses a primary phenomenon for the generation of a dominant hypothesis, and based on that hypothesis, seeks secondary experiments/phenomena for further validation; the logical reasoning structure is essentially linear and in series. The decentralized approach uses a broad suite of primary phenomena, accumulated without preconception of any kind, for the simultaneous derivation of many hypotheses; the logical reasoning structure is reticular and in parallel.
