# Supplementary material for: A Decentralized Approach to the Formulation of Hypotheses: A Hierarchical Structural Model for a Prion Self-Assembled System
Source: Sci Rep. 2016 Jul 28;6:30633. doi: 10.1038/srep30633 (PMC4964355; doi:10.1038/srep30633)
Supplement: Supplementary Information [file srep30633-s1.pdf]

Supplementary Information for

A Decentralized Approach to the Formulation of Hypotheses: A Hierarchical Structural Model for a Prion Self-Assembled System

Mingyang Wang, Feifei Zhang, Chao Song, Pengfei Shi & Jin Zhu*

Department of Polymer Science and Engineering, School of Chemistry and Chemical Engineering, State Key Laboratory of Coordination Chemistry, Nanjing National Laboratory of Microstructures, Collaborative Innovation Center of Chemistry for Life Sciences, Nanjing University, Nanjing 210093, China

**Corresponding author. Phone: +86-25-8968-6291; Fax: +86-25-8331-7761; Email: jinz@nju.edu.cn*

TABLE OF CONTENTS

Figure S1. pET28a-Sup35-NM vector and induced expression of Sup35-NM protein.

Figure S2. Protein purification.

Figure S3. Congo red binding results for Sup35-NM fibrils.

Figure S4. TEM of Sup35-NM FP and AG fibrils.

Figure S5. Effect of stirring on the Sup35-NM self-assembly process as monitored by Congo red binding experiment.

Figure S6. BN-PAGE for 2-month AG fibrils under different treating conditions.

Figure S7. Effect of stirring on the appearance of ATmer within 7 days.

Figure S8. SEC curves for FP and AG fibrils in the absence and presence of 2% SDS.

Figure S9. Calculation method for the SEC eluted percentage.

Table S1. The percentage of 41 min species at 20 h over total protein in the Sup35-NM fibril growth process.

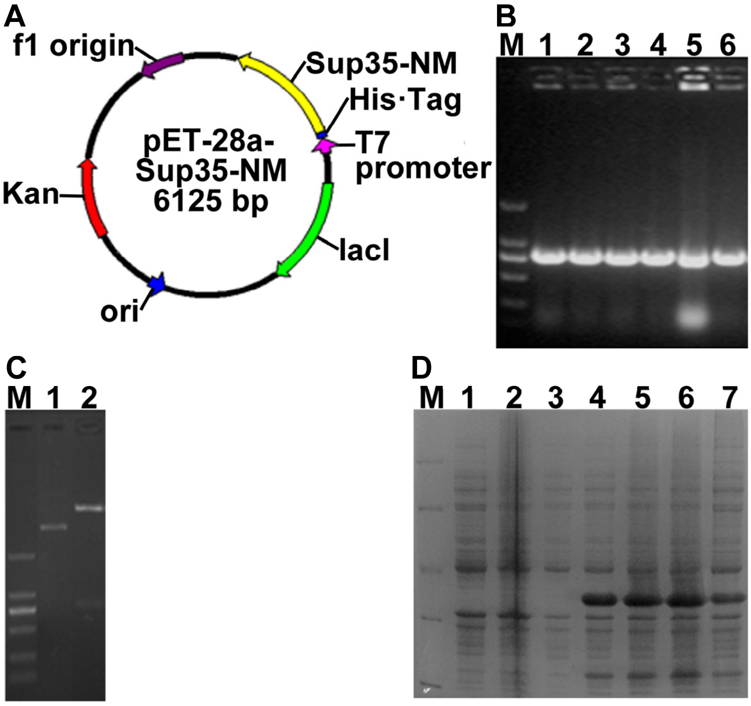

Figure S1. pET28a-Sup35-NM vector and induced expression of Sup35-NM protein. (A) Diagram of pET28a-Sup35-NM vector. (B) PCR detection of pET28a-Sup35-NM vector in BL21 DE3 (Rosseta). Lanes 1-6: different transformants. (C) The restriction enzyme digestion of pET28a-Sup35-NM vector. Lane 1: circular pET28a-Sup35-NM vector; lane 2: pET28a-Sup35-NM vector after digestion by Not I and Nde I restriction enzymes. (D) Effect of induction times on Sup35-NM expression in BL21 DE3 (Rosseta) cells with vectors pET28a (lanes 1-3: 2 h, 4 h, 6 h) and pET28a-Sup35-NM (lanes 4-6: 2 h, 4 h, 6 h). Lanes M in Figures S1B and S1C correspond to DL 2000 (Takara). Lane M in Figure S1D was for unstained protein molecular weight marker (Thermo, 26610), corresponding to 116 kDa, 66.2 kDa, 45 kDa, 35 kDa, 25 kDa.

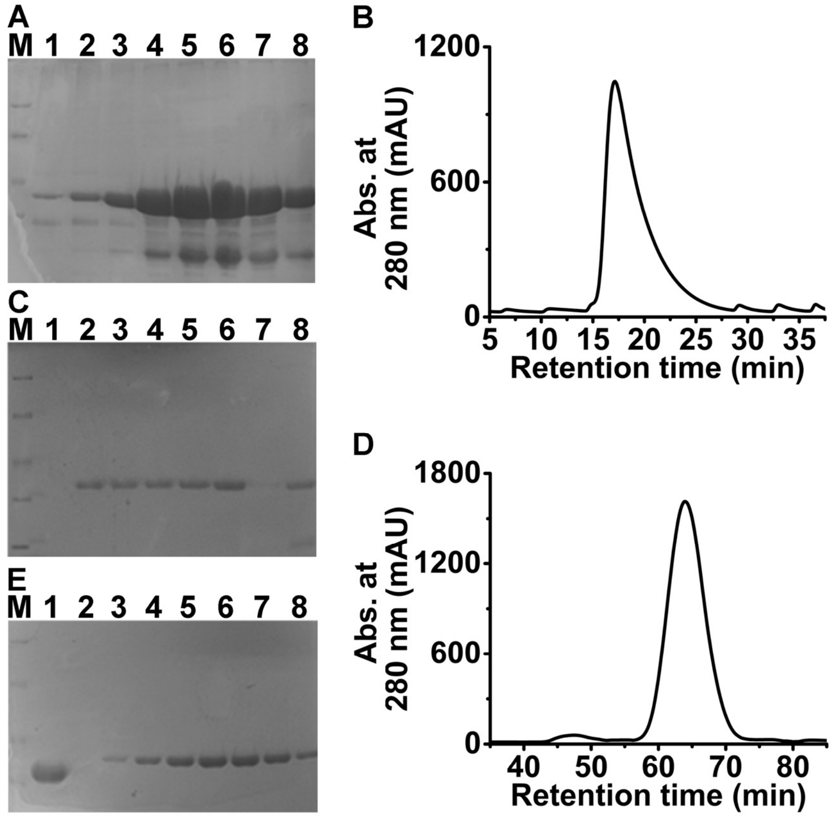

Figure S2. Protein purification. (A) SDS-PAGE for Ni^{2+}-NTA column eluted Sup35-NM protein. (B) Elution curve for anion exchange resin (Q HP) in the second purification stage. (C) SDS-PAGE of the fractionated samples in Figure S2B. The main peak was eluted at 50% gradient. Lanes 1-7: samples with different retention times (spanning approximately 15-22 min) in the peak; lane 8: sample before purification with anion exchange. (D) SEC curve of eluted protein. The 64 min peak represented the denatured Sup35-NM monomer. (E) SDS-PAGE of the fractionated samples in Figure S2D. Lane 1: sample before purification with SEC; lanes 2-7: samples with different retention times in the 64 min peak. The molecular weight marker for lanes M in Figures S2A, 2C, and 2E was the same as that used in Figure S1D, lane M.

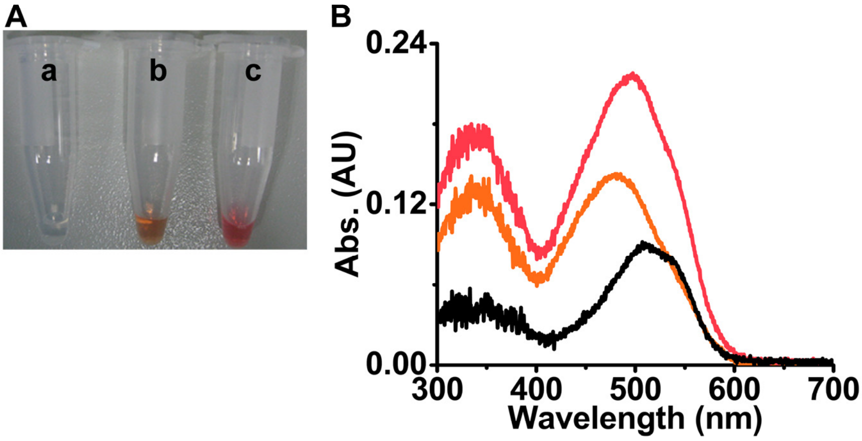

Figure S3 Congo red binding results for Sup35-NM fibrils. (A) Solutions (in buffer C) of Sup35-NM fibrils (a), Congo red (b) and Congo red-bound fibrils (c). (B) Absorption curves for Congo red (10 μM) (orange) and Congo red-bound Sup35-NM fibrils (1 μM) (red). The difference spectrum showed a peak spanning approximately 400 to 600 nm.

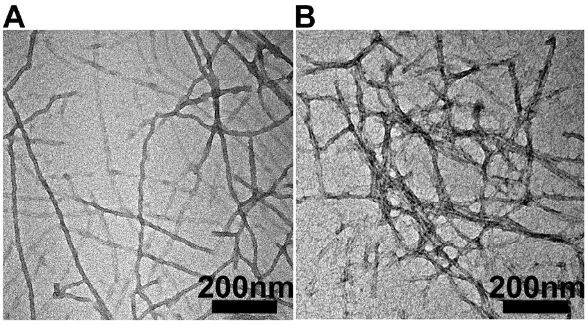

Figure S4. TEM of Sup35-NM FP (A) and AG (B) fibrils. The sample of Sup35-NM FP fibrils used was 20 μM (suspended in buffer C).

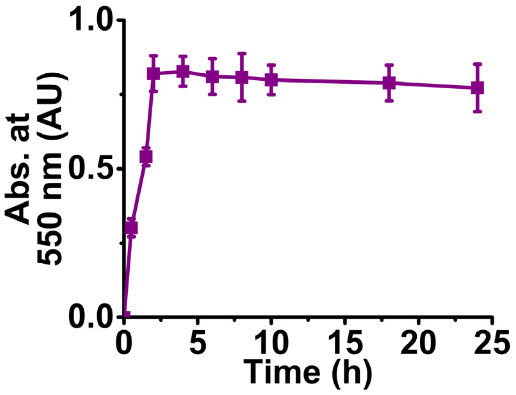

Figure S5. Effect of stirring on the Sup35-NM self-assembly process as monitored by Congo red binding experiment. The final protein concentration of Sup35-NM reacting with Congo red was 10 μM (in buffer C).

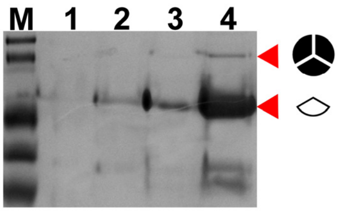

Figure S6. BN-PAGE for 2-month AG fibrils (20 μM) under different treating conditions. Lane 1: 2-month AG fibrils; lanes 2-4: 2-month AG fibrils under SDS, boiling, and SDS-boiling conditions, respectively. The molecular weight marker for lane M was the same as that used in Figure S1D, lane M.

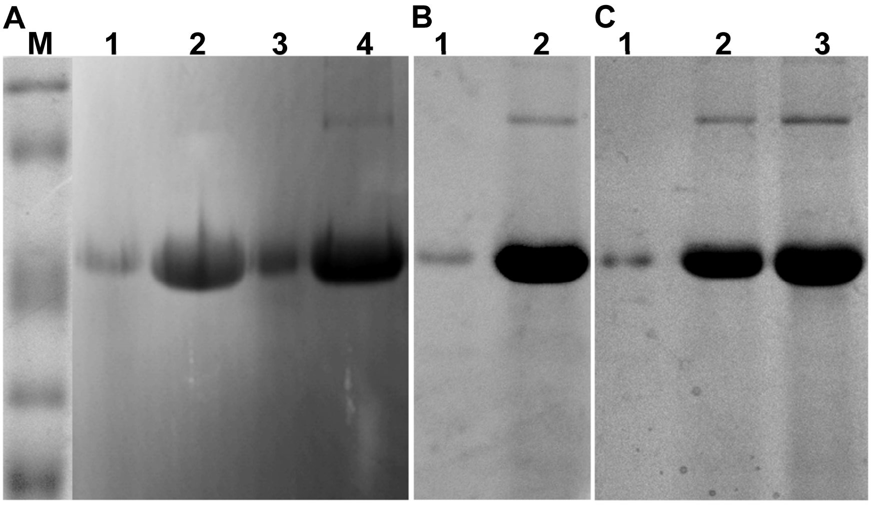

Figure S7. Effect of stirring on the appearance of ATmer within 7 days. (A) BN-PAGE of 2-day ST fibrils formed from desalted monomer (40 μM). Fibrils prepared without stirring (lane 1: directly loaded; lane 2: under 4% SDS-boiling condition); ST Fibrils (lane 3: directly loaded; lane 4: under 4% SDS-boiling condition). (B) BN-PAGE of 4-day ST fibrils. Lane 1: directly loaded; lane 2: under 4% SDS-boiling condition. (C) BN-PAGE of 7-day ST fibrils. Lane 1: directly loaded; lane 2: under 4% SDS-65 °C condition; lane 3: under 4% SDS-boiling condition. The molecular weight marker for lane M was the same as that used in Figure S1D, lane M.

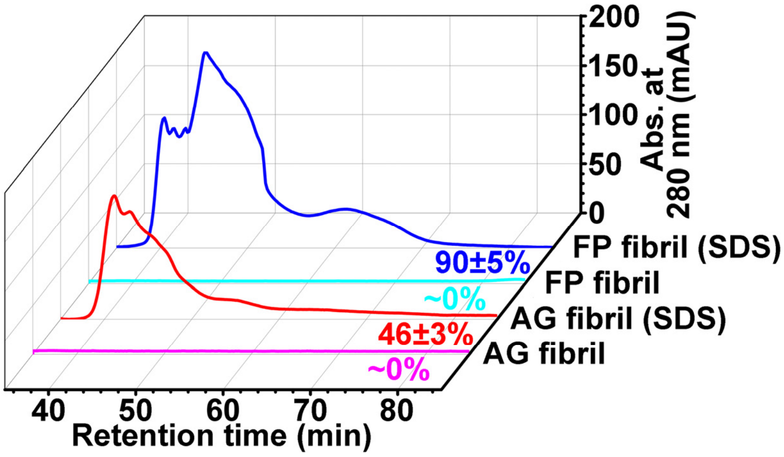

Figure S8. SEC curves for FP and AG fibrils in the absence (cyan and pink) and presence (blue and red) of 2% SDS. The concentration of all samples used was 20 μM (in buffer C). The percentage of eluted portion of protein over the total protein was shown under each curve.

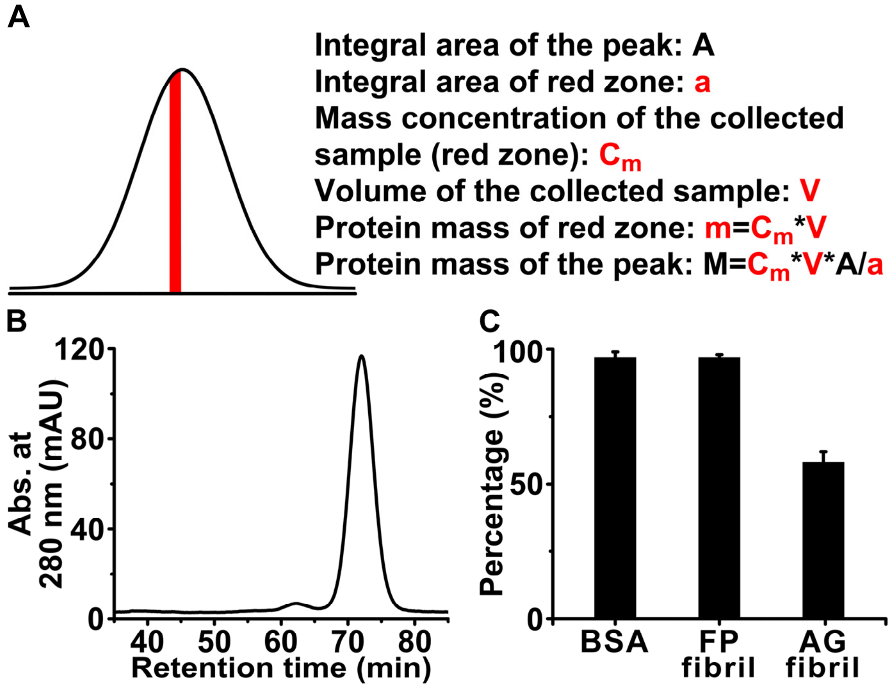

Figure S9. Calculation method for the SEC eluted percentage. (**A**) Calculation method for eluted quantity of protein associated with a peak. (**B**) The albumin curve (Pierce, product #23209, 2 mg/mL) in SEC. The volume of albumin used was 0.5 mL. Albumin was used as a reference for evaluating the accuracy of the calculation method described in Figure S9A. (**C**) Percentage of eluted BSA, and FP and AG fibrils under SDS-boiling condition. The eluted percentage of albumin by SEC was calculated to be 97%, indicating that the calculation was reliable.

Table S1. The percentage of 41 min species at 20 h over total protein in the Sup35-NM fibril growth process. Samples 1, 2, and 3 represented three independent experiments.

Sample	41 min species integration area	Total integration area	41min/Total (%)
1	469.5	545.8	86.0
2	452. 2	572.8	78.9
3	652.4	778.1	83.8
